# Association of TMEM106B with Cortical *APOE* Gene Expression in Neurodegenerative Conditions

**DOI:** 10.3390/genes15040416

**Published:** 2024-03-26

**Authors:** Cynthia Picard, Justin Miron, Judes Poirier

**Affiliations:** 1 Douglas Mental Health University Institute, Montreal, QC H4H 1R3, Canada; cynthia.picard@mail.mcgill.ca (C.P.); justin.miron@mail.mcgill.ca (J.M.); 2 Centre for the Studies on Prevention of Alzheimer’s Disease, Montreal, QC H4H 1R3, Canada; 3 Department of Psychiatry, Faculty of Medicine, McGill University, Montreal, QC H3A 0E7, Canada

**Keywords:** TMEM106B, APOE, Alzheimer’s disease, neurodegeneration, ECL mouse model, gene regulation, trans-eQTL analysis, cortical gene expression

## Abstract

The e4 allele of the apolipoprotein E gene is the strongest genetic risk factor for sporadic Alzheimer’s disease. Nevertheless, how *APOE* is regulated is still elusive. In a *trans*-eQTL analysis, we found a genome-wide significant association between transmembrane protein 106B (*TMEM106B*) genetic variants and cortical *APOE* mRNA levels in human brains. The goal of this study is to determine whether TMEM106B is mis-regulated in Alzheimer’s disease or in other neurodegenerative conditions. Available genomic, transcriptomic and proteomic data from human brains were downloaded from the Mayo Clinic Brain Bank and the Religious Orders Study and Memory and Aging Project. An in-house mouse model of the hippocampal deafferentation/reinnervation was achieved via a stereotaxic lesioning surgery to the entorhinal cortex, and mRNA levels were measured using RNAseq technology. In human temporal cortices, the mean TMEM106B expression was significantly higher in Alzheimer’s disease compared to cognitively unimpaired individuals. In the mouse model, hippocampal *Tmem106b* reached maximum levels during the early phase of reinnervation. These results suggest an active response to tissue damage that is consistent with compensatory synaptic and terminal remodeling.

## 1. Introduction

The apolipoprotein E e4 allele differs from e3 by a cysteine to arginine change at position 112. Early studies showed a significant association between e4 and sporadic Alzheimer’s disease, and this association was more pronounced in women [1,2]. To date, *APOE* is still the strongest genetic risk factor for late-onset AD, although the precise mechanisms governing the *APOE* expression remain unclear. Whether AD patients exhibit an increased or decreased *APOE* expression, and whether there are reasons for the varying correlations between APOE mRNA and protein levels across different studies, remains ambiguous [3].

Due to these factors, we chose to examine *APOE* mRNA levels in Alzheimer’s disease-affected brains. By performing a *trans*-eQTL analysis, we identified a genome-wide significant locus in the *TMEM106B* gene region. Our main focus was on TMEM106B and examining how its regulation is altered in AD and other neurodegenerative conditions.

TMEM106B is a highly glycosylated transmembrane protein located in endosomes and lysosomal membranes [4]. TMEM106B controls the size, number, motility, trafficking and acidification of lysosomes [5]. In neurons, increasing TMEM106B levels result in the enlargement of the lysosomal compartment, without affecting the lysosome number [6]. The suppression of TMEM106B shows a significant decrease in the lysosome number and inhibition of dendritic branching [6,7]. When the retrograde transport is inhibited via the interaction of TMEM106B with MAP6, the anterograde transport through microtubules is facilitated [8].

Neurons, with their large size and polarized structure, face distinctive challenges when it comes to maintaining cellular homeostasis in regions distant from the cell body, where mature lysosomes are enriched. To guarantee the effective elimination of cellular waste in distant axonal areas, neurons depend on tightly synchronized bidirectional transport within the cell. Impaired lysosomal transport results in progressive neurodegeneration in most lysosomal storage diseases (LSDs) and contributes to the pathogenesis of age-related neurodegenerative diseases [9].

In mucolipidosis type IV, a lysosomal storage disease, the changes observed in microglia exhibit similarities with alterations found in typical neurodegenerative conditions like Alzheimer’s, Parkinson’s, and Huntington’s diseases [10]. The accumulation of inert substrates has been considered as the primary factor responsible for the pathology and clinical symptoms observed in LSDs. Considering the crucial role of lysosomes in cellular homeostasis and metabolism, it has been speculated that impaired storage is just the “instigator” of a host of secondary events [11].

These events consist of the secondary storage of unrelated substances, abnormal composition of membranes, aberrant fusion and intracellular trafficking of vesicles, altered autophagy, mitochondrial dysfunction, oxidative stress and inflammation [12]. Stored materials can disrupt ligand–receptor interactions, alter receptor responses, and impact the internalization and recycling of receptors, resulting in the modified activation of signaling pathways related to cellular transport, calcium balance and inflammation as well as cell death [10].

Another example of LSD is the Niemann-Pick type C disease (NPC), characterized by progressive neurodegeneration. NPC nerve cells demonstrate not only the aberrant storage of cholesterol but also neurofibrillary tangles (NFT), which are typically found in Alzheimer’s disease, Down syndrome and progressive supranuclear palsy [13]. Inappropriate storage, in the form of inclusions, are often marked with ubiquitin-positive proteins. It is thought that lysosomal deposits might derive from an unsuccessful attempt at elimination by autophagy [14].

Ubiquitin-immunoreactive nuclear inclusions are detected in neurons in Huntington’s disease and spinocerebellar ataxias [15]. Most other ubiquitin-positive inclusions are found in the cytoplasm in neurodegenerative disorders such as Alzheimer’s disease, amyotrophic lateral sclerosis, diffuse Lewy body disease, frontotemporal dementia, Parkinson’s disease, Pick’s disease and progressive supranuclear palsy [14,16].

Our initial findings showed a strong correlation between *TMEM106B* genetic variants and *APOE* mRNA levels, which suggest an involvement in Alzheimer’s disease. To better understand the role of TMEM106B in neurodegenerative conditions, we measured its expression levels in available human brain tissues affected with different neuropathologies and in the well-established mouse model of a hippocampal deafferentation in response to entorhinal cortex lesioning (ECL).

## 2. Materials and Methods

### 2.1. ROSMAP Cohort

The Religious Orders Study and Memory and Aging Project (ROSMAP) is a longitudinal clinical–pathologic cohort study of aging and Alzheimer’s disease. Participants were enrolled from more than 40 groups of religious orders (nuns, priests, brothers, etc.) across the United States. The enrollment required no known signs of dementia. Medical conditions were documented since 1994 with clinical evaluations or self reports. The Alzheimer’s disease status was determined by a computer algorithm based on a cognitive test performance with a series of discrete clinical judgments made by a neuropsychologist and clinician. Individuals were categorized as cognitively unimpaired (CU) if they were diagnosed without dementia or mild cognitive impairment (MCI). Diagnoses of dementia and Alzheimer’s disease conform to standard definitions. A clinician reviewed all cases determined by this algorithm. Upon death, a post-mortem neuropathologic evaluation that includes a uniform structured assessment of Alzheimer’s disease pathology, cerebral infarcts, Lewy body disease and other pathologies common in aging and dementia is performed. The ROS and MAP studies are described in detail in these two articles from 2012 [17,18] (see Table S1 for demographics). Subjects’ consent was obtained according to the Declaration of Helsinki.

#### 2.1.1. *APOE* and *TMEM106B* Expression Levels from Human Dorsolateral Prefrontal Cortices

Gene expression data were downloaded from AMP-AD Knowledge Portal https://adknowledgeportal.synapse.org (synapse ID: syn3800853, accessed on 15 February 2022). 

Total RNA was extracted using the Rneasy lipid tissue kit (Qiagen, Valencia, CA, USA). The mean ± SD for sample RNA Integrety Number was 6.8 ± 0.8.

Custom microarrays manufactured by Agilent Technologies were used with mRNA samples from the ROSMAP study described above. Microarrays consisted of 4720 control probes and 39,579 probes targeting transcripts representing 25,242 known and 14,337 predicted genes. The gene expression was reported as the mean-log ratio of individual microarray intensities relative to average intensities of all samples [19] (see Table S1 for demographics). The consent from the subjects was acquired in accordance with the Declaration of Helsinki.

#### 2.1.2. APOE and TMEM106B Protein Levels from Human Dorsolateral Prefrontal Cortices

Proteomics data were downloaded from AMP-AD Knowledge Portal https://adknowledgeportal.synapse.org (synapse ID: syn17008935, accessed on 15 February 2022). Tandem Mass Tag (TMT) isobaric labeling and synchronous precursor selection-based MS3 (SPS-MS3) mass spectrometry were performed on dorsolateral prefrontal cortices (DLPFC) from the ROSMAP proteomic study [20].

#### 2.1.3. Genotype Data from ROSMAP

Genotype data were obtained from AMP-AD Knowledge Portal https://adknowledgeportal.synapse.org (synapse ID: syn3157325, accessed on 15 February 2022) through downloading.

gDNA was extracted from 1709 individuals from the ROSMAP study and analyzed using the Affymetrix GeneChip 6.0 (Affimetrix, Inc., Santa Clara, CA, USA) at the Broad Institute’s Center for Genotyping or the Translational Genomics Research Institute. Only individuals with European ancestry were genotyped to minimize the population heterogeneity. Subjects’ consent was obtained following the Declaration of Helsinki.

A sample-level quality control assessment included the exclusion of samples with a genotype success rate < 95%, discordance between the inferred and reported gender, and excess inter/intraheterozygosity. The SNP-level quality control assessment included the exclusion of SNPs with a deviation from the Hardy-Weinberg equilibrium (*p* < 0.001), minor allele frequency <0.01 and genotype call rate <0.95. Missing genotypes were obtained via an imputation method.

The imputation was performed by Sanger Imputation Service using 1000 Genomes (phase 3) as the reference panel [21]. Pre-phasing was performed with SHAPEIT2 and PBWT [22,23]. Only post-imputed SNPs with an info score >0.7 were kept. A total of 10 324,516 variants passed the quality control and 5,007,676 were left after applying a minor allele frequency threshold of 5%.

### 2.2. The Mayo Clinic Cohort

Gene expression data were downloaded from AMP-AD Knowledge Portal https://adknowledgeportal.synapse.org (synapse ID: syn5550404, accessed on 15 February 2022). 

Total RNA was extracted from frozen brain samples using the Ambion RNAqueous kit (Life Technologies, Grand Island, NY, USA) according to manufacturer’s instructions. The quantity and quality of all RNA samples were determined by the Agilent 2100 Bioanalyzer using the Agilent RNA 6000 Nano Chip (Agilent Technologies, Santa Clara, CA, USA). Samples had to have an RNA Integrity Number ≥ 5.0 for inclusion in the study.

Gene expression measures were generated, using next-generation RNA-sequencing (RNAseq), from temporal cortex RNA samples collected for 266 subjects from the Mayo Clinic Brain Bank and Banner Sun Health research institute [24]. These 266 subjects have the following pathological diagnoses: Alzheimer’s disease (AD; *n* = 80), progressive supranuclear palsy (PSP; *n* = 82), pathological aging (PA; *n* = 30) and cognitively unimpaired (CU; *n* = 74).

Control subjects each had a Braak NFT stage of 3.0 or less, CERAD neuritic and cortical plaque densities of 0 (none) or 1 (sparse) and lacked any of the following pathological diagnoses: Alzheimer’s disease, Parkinson’s disease, Lewy body disease, vascular dementia, progressive supranuclear palsy, motor neuron disease, corticobasal degeneration, Pick’s disease, Huntington’s disease, frontotemporal lobar degeneration, hippocampal sclerosis or dementia lacking distinctive histology. Subjects with pathological aging also lacked the above diagnoses and had a Braak NFT stage of 3.0 or less but had CERAD neuritic and cortical plaque densities of 2 or more. None of the pathologic aging subjects had a clinical diagnosis of dementia or mild cognitive impairment. Given the presence of amyloid plaques, but not tangles and the absence of dementia, pathologic aging is considered to be either a prodrome of Alzheimer’s disease or a condition, in which there is resistance to the development of NFT and/or dementia [24,25].

All subjects were North American Caucasians and all of them had ages at death ≥ 58 (see Table S1 for demographics). Consent from the subjects was obtained in adherence to the principles outlined in the Declaration of Helsinki.

### 2.3. Animals

Two-to-three-month-old male C57BL/6J wild-type mice were purchased from Jackson Laboratories (Bar Harbor, ME, USA). All animals were housed individually in an enriched environment and fed ad libitum with a standard laboratory chow diet. A 12 h light–dark cycle was maintained with light onset at 7:00 and offset at 19:00, local time. All protocols were carried out in accordance with the Canadian Guidelines for Use and Care of Laboratory Animals and were approved by the McGill University Animal Care Committee.

#### 2.3.1. Unilateral Entorhinal Cortex Lesions (ECL)

Unilateral electrolytic lesions to the entorhinal cortex were performed on six mice per time point, as described by Blain et al. [26]. Mice were anaesthetized with isoflurane and placed in a stereotaxic apparatus in a flat skull position. Four lesion coordinates were determined from Lambda: (1) [AP: 0 mm], [L: −3 mm] and [DV: −3 mm, −4 mm]; (2) [AP: 0 mm], [L: −3.5 mm] and [DV: −3 mm, −4 mm]; (3) [AP: +0.5 mm], [L: −4 mm] and [DV: −3 mm, −4 mm]; (4) [AP: +1 mm], [L: −4 mm] and [DV: −3 mm, −4 mm]. A 1 mA current was applied for 10 s at each coordinate. Six sham-operated animals were treated similarly except that the electrode was applied without a current and lowered only by 1 mm.

Following surgery, a subcutaneous booster of physiological saline was injected into mice to prevent dehydration. Analgesic buprenorphine (0.1 mg/Kg) was administered 3 times a day (prior to waking up, and every 4–8 h) and then at 24, 48 and 72 h. Mice were monitored daily for possible adverse side effects such as weight loss or dyskinesia. Mice were sacrificed by CO_2_ asphyxiation under isoflurane anesthesia after 2, 7, 14, 21 and 40 days post lesion (DPL). Sham-lesioned mice were all sacrificed on the same day. Mice were decapitated, and their brains were quickly removed. Contralateral and ipsilateral hippocampi were dissected on dry ice and stored at −80 °C until use.

#### 2.3.2. RNA Extraction from Mice Hippocampi

The total RNA was extracted from pools of two hippocampi using the RNeasy Lipid Tissue Mini Kit (Qiagen, Hilden, Germany). The RNA quality was assessed by the McGill University and Génome Québec Innovation Centre. All samples had an RNA integrity number greater than 7.8 and a 260/280 ratio greater than 2.1.

#### 2.3.3. *Tmem106b* Expression Levels in ECL Mice

*Tmem106b* mRNA levels were measured with the Mouse Clariom^TM^ D Assay (Affymetrix, Santa Clara, CA, USA) by the McGill University and Génome Québec Innovation Centre. The resulting CEL files were analyzed with the Transcriptome Analysis Console (TAC) Version 4.0 using the Mouse Transcriptome Assay (MTA) 1.0 Array. TAC 4.0 uses the Limma Bioconductor package for an expression analysis which performs ANOVA with empirical Bayesian corrections [27].

### 2.4. Statistical Analyses

**Figure 1:**
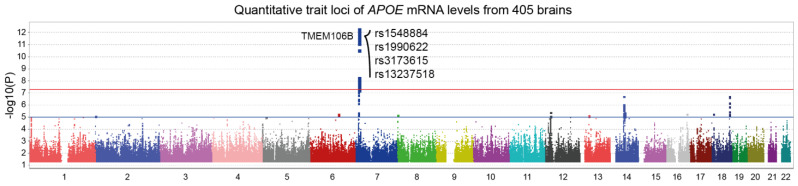
Manhattan plot of *APOE* expression quantitative trait loci (eQTL). *APOE* mRNA levels from 405 dorsolateral prefrontal cortices were made available by ROSMAP using custom microarrays manufactured by Agilent Technologies. *Trans*-eQTL analysis revealed a genome-wide significant locus in *TMEM106B* gene region (*p* = 4.4 ×10^−13^). Top SNPs are indicated on the graph. Suggestive (blue) and genome-wide significant (red) thresholds were set to a -log10 *p* value of 5 and 7.3, respectively.

**Figure 2:**
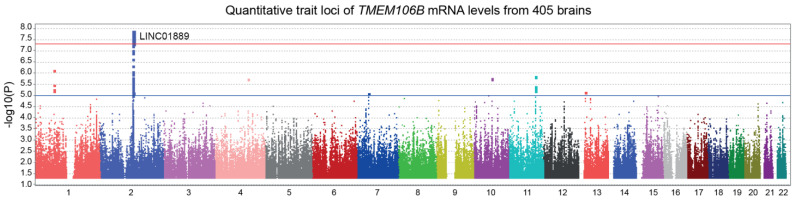
Manhattan plot illustrating the expression quantitative trait loci (eQTL) of *TMEM106B*. Custom microarrays manufactured by Agilent Technologies were used to measure *TMEM106B* mRNA levels from 405 dorsolateral prefrontal cortices from the ROSMAP cohort. *A* genome-wide significant locus in *LINC01889* gene region was found by the *trans*-eQTL analysis (*p* = 1.3 × 10^−8^). Suggestive (blue) and genome-wide significant (red) thresholds were set to a -log10 *p* value of 5 and 7.3, respectively.

**Figure 3:**
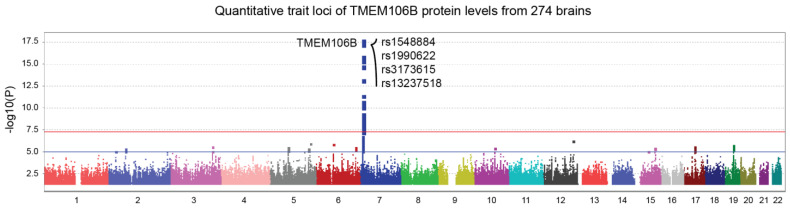
TMEM106B protein quantitative trait loci (pQTL) represented by a Manhattan plot. TMEM106B protein levels from 274 dorsolateral prefrontal cortices were made available by ROSMAP using tandem mass tag proteomics. The Pan-genomic analysis revealed a genome-wide significant locus in the *TMEM106B* gene region (*p* = 1.5 × 10^−18^). Top SNPs are shown on the graph. Suggestive (blue) and genome-wide significant (red) thresholds were set to a -log10 *p* value of 5 and 7.3, respectively.

**Figure 4:**
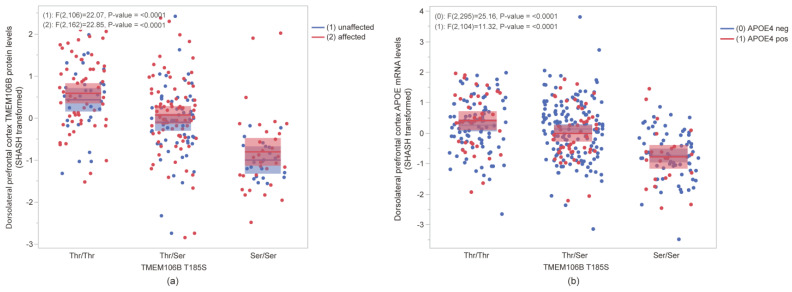
Influence of TMEM106B coding variant T185S in the dorsolateral prefrontal cortex from ROSMAP individuals. (**a**) TMEM106B protein levels were significantly reduced in the presence of 185S amino acid (*p* < 0.001) in both unaffected (blue) and affected (red) individuals. (**b**) In both *APOE4* negative (blue) and *APOE4* positive (red) individuals, *APOE* mRNA levels were significantly lower in the Ser/Ser group (*p* < 0.001). Mean ± SEM are represented by shaded area of corresponding colors for each group.

**Figure 5:**
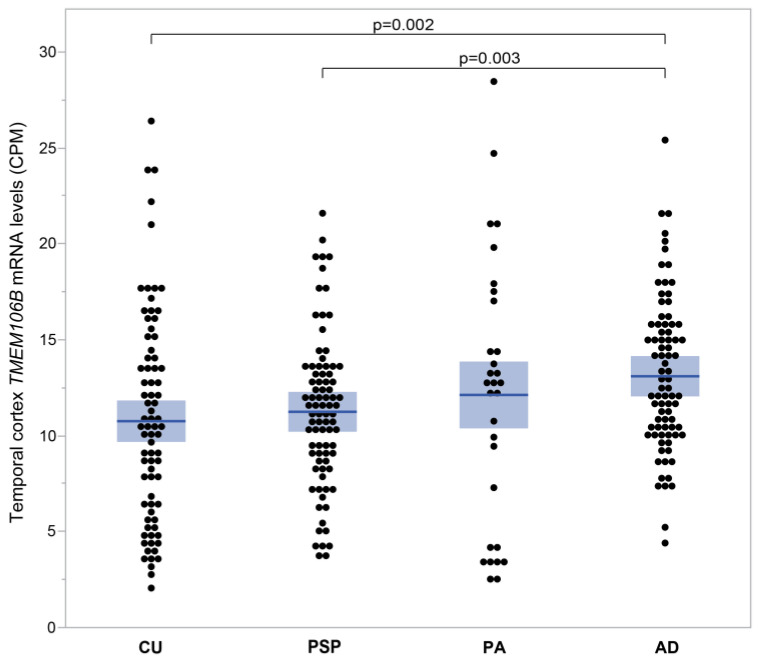
*TMEM106B* mRNA levels measured in human temporal cortices affected with different neuropathologies. *TMEM106B* mRNA levels were measured by RNAseq in the Mayo Clinic cohort. Mean ± SEM (shaded area) are presented for four groups of individuals, each with a different diagnostic. *TMEM106B* mean expression increased progressively from CU (10.76 ± 0.62 CPM; *n* = 74), to PSP (11.25 ± 0.43 CPM; *n* = 82), PA (12.13 ± 1.27 CPM; *n* = 30) and finally AD (13.10 ± 0.44 CPM; *n* = 80). *TMEM106B* mRNA levels were elevated in AD compared to CU (*p* = 0.002) and PSP (*p* = 0.003).

**Figure 6:**
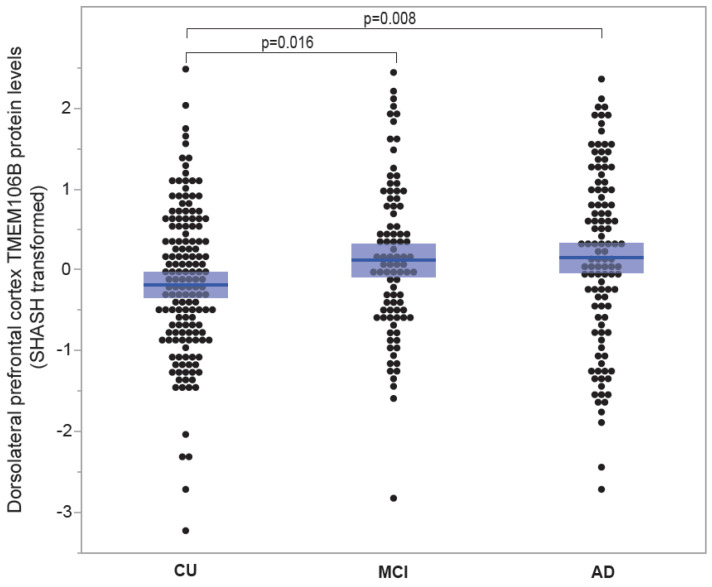
TMEM106B protein levels according to disease status. TMEM106B protein levels from the ROSMAP cohort were measured using tandem mass tag proteomics. Mean ± SEM (shaded area) are presented for three groups classified by disease. Mean TMEM106B levels increased from CU (−0.181 ± 0.074; *n* = 152), to MCI (0.122 ± 0.102; *n* = 90) and AD (0.152 ± 0.104; *n* = 110). TMEM106B protein levels were significantly higher in MCI compared to CU (*p* = 0.016) and significantly higher in AD compared to CU (*p* = 0.008).

In Figure 1, Figure 2 and Figure 3, genetic association analyses were performed with PLINK [28]. For Figure 4 and Figure S2, JMP Pro 17 was used to perform a logistic regression between the different genotypes. Unpaired two-tailed t-tests were used to compare the TMEM106B expression or protein levels between 2 groups (Figure 5 and Figure 6). For Figure 4 and Figure 6, SHASH transformed data were obtained with JMP Pro 17 and were normally distributed according to the Anderson-Darling test. The LD plot (Figure S1) and corresponding values were calculated and visualized using the Haploview software version 4.2 from the Broad Institute [29]. JMP Pro 17 was also used to perform a linear regression in Figure S3. For the *Tmem106b* expression in the ECL mouse model, unpaired two-tailed t-tests were used to compare each time point against sham-operated animals (Figure S4).

## 3. Results

### 3.1. Human Brains

#### 3.1.1. *APOE* mRNA Levels Are Strongly Influenced by TMEM106B Genetic Variants

Figure 1 illustrates a *trans*-eQTL analysis of *APOE* mRNA levels measured in 405 dorsolateral prefrontal cortices from ROSMAP subjects. A genome-wide significant locus was found on chromosome 7, corresponding to the *TMEM106B* gene region. Interestingly, four of the top SNPs are found in the literature in association with various medical conditions such as Alzheimer’s disease, frontotemporal dementia, amyotrophic lateral sclerosis, chronic traumatic encephalopathy, Parkinson’s disease and hippocampal sclerosis (Table 1). These results also suggest that *APOE* is either co-regulated with *TMEM106B* or influenced by TMEM106B protein levels. To test these hypotheses, we performed eQTL and pQTL analyses on TMEM106B mRNA and protein levels, respectively.

#### 3.1.2. *TMEM106B* mRNA Levels Are not Influenced by Cis-Acting Polymorphisms

In Figure 2, we performed a *trans*-eQTL analysis of *TMEM106B* mRNA levels measured in 405 dorsolateral prefrontal cortices from the ROSMAP cohort. The *LINC01889* gene region, found on chromosome 2, reached a genome-wide significance. LINC01889 is a long intergenic non-protein coding RNA of an unknown function. No significant *cis*-acting SNPs were found in association with *TMEM106B* mRNA levels. Therefore, it is very unlikely that *APOE* and *TMEM106B* genes are regulated similarly.

#### 3.1.3. TMEM106B Protein Levels Are Influenced by Nearby Genetic Variants

Pan genomic pQTL analysis using TMEM106B protein levels from the ROSMAP cohort revealed a genome-wide significant locus in the *TMEM106B* gene region (Figure 3, top SNP; *p* = 1.5 × 10^−18^). Here, the same SNPs from Figure 1 exhibited an even more robust correlation with TMEM106B protein levels. These results show that SNPs influencing TMEM106B protein levels also affect *APOE* gene regulation, through a yet unknown mechanism. The four SNPs from Figure 1 and Figure 3 are in a strong linkage disequilibrium, as shown in Figure S1. Rs3173615 is a coding variant of the *TMEM106B* gene, where a threonine is replaced with a serine at position 185.

#### 3.1.4. The T185S Coding SNP Correlates with Lower TMEM106B Protein Levels and Lower *APOE* mRNA Levels

To assess the impact of the T185S TMEM106B coding SNP discussed above (rs3173615), we used targeted proteomic and microarray results from the ROSMAP cohort. In both affected (MCI and AD) and unaffected (CU) individuals, the T185S variant correlated strongly (*p* = 2.26 × 10^−18^; *n* = 274) with reduced cortical TMEM106B protein levels (Figure 4a). Furthermore, T185S correlated strongly (*p* = 4.79 × 10^−13^; *n* = 405) with reduced *APOE* mRNA levels, regardless of *APOE4* genotype (Figure 4b). Of note, APOE protein levels were not affected by the T185S amino acid change (Figure S2). A strong association was found between TMEM106B protein levels and *APOE* mRNA levels (*p* = 8.72 × 10^−8^; *n* = 107), regardless of the *APOE4* genotype (Figure S3). Since TMEM106B seems to act upstream of *APOE,* next, we ask whether TMEM106B is actually misregulated in the brain in AD or in other neurodegenerative conditions.

#### 3.1.5. *TMEM106B* mRNA Levels Are Elevated in AD Temporal Cortices Compared to Brain Tissues from Cognitively Unimpaired Individuals

Figure 5 compares mRNA expression levels of *TMEM106B* between cognitively unimpaired individuals (CU) and individuals with progressive supranuclear palsy (PSP), pathological aging (PA) and Alzheimer’s disease (AD) from the Mayo clinic cohort. Figure 5 illustrates the mean *TMEM106B* expression as it increases progressively from CU (10.76 ± 0.62 CPM; *n* = 74), to PSP (11.25 ± 0.43 CPM; *n* = 82), PA (12.13 ± 1.27 CPM; *n* = 30) and finally AD (13.10 ± 0.44 CPM; *n* = 80). *TMEM106B* mRNA levels are significantly higher in AD compared to CU (*p* = 0.002) and PSP (*p* = 0.003).

To further investigate whether TMEM106B is involved in the clinical progression of Alzheimer’s disease, we turned to the cross-sectional ROSMAP cohort, which includes cognitively unimpaired individuals and individuals with mild cognitive impairment (MCI) and Alzheimer’s disease (AD).

#### 3.1.6. TMEM106B Protein Levels Are Elevated in Individuals Affected with Mild Cognitive Impairment and Alzheimer’s Disease Compared to Cognitively Unimpaired Individuals

Figure 6 contrasts cortical TMEM106B protein levels between cognitively unimpaired individuals and individuals affected with MCI and AD. Average TMEM106B levels exhibited an increment from CU (−0.181 ± 0.074; *n* = 152), to MCI (0.122 ± 0.102; *n* = 90) and AD (0.152 ± 0.104; *n* = 110). TMEM106B protein levels were notably elevated in MCI compared to CU (*p* = 0.016) and significantly higher in AD compared to CU (*p* = 0.008).

### 3.2. Animals

#### *Tmem106b* Levels Are Reduced during the Hippocampal Deafferentation Phase and Reach Peak Levels during the Reinnervation Phase in Entorhinal Cortex Lesioned Mice

In mice, the hippocampus and cerebellum are the brain regions with high levels of *Tmem106b* expression (Allen Mouse Brain Atlas, https://mouse.brain-map.org/static/atlas); thus, these two regions might be the most susceptible to alterations of the TMEM106B function. Entorhinal cortex lesioning (ELC) is a well-described model of the anterograde axonal degeneration, followed by sprouting and compensatory reactive synaptogenesis in the hippocampus [47]. Hippocampal neurons display a transient reduction of synaptophysin immunoreactivity between 7 to 14 days post lesion (DPL) during the acute phase of deafferentation, followed by a normalization of the protein levels at 21 DPL in response to the compensatory synaptogenesis in the deafferented zone of the outer molecular layer [26,47]. The use of unilateral lesions offers an experimental benefit by allowing the simultaneous generation of a contralateral, unlesioned hemisphere that serves as a negative control for each animal under investigation.

In Figure S4, ipsilateral hippocampi showed reduced *Tmem106b* expression levels in the deafferentation phase (DPL2: *p* = 0.046; *n* = 6) and reached maximum levels during the early phase of reinnervation (DPL14: *p* = 0.030; *n* = 6 and DPL21: *p* = 0.019; *n* = 6), compared to sham-operated animals (*n* = 6). These results are concordant with the retrograde transport being used to remove damaged organelles and the subsequent sprouting and compensatory synaptogenesis being facilitated by the anterograde transport [8,48].

## 4. Discussion

In 2010, an international collaboration identified three SNPs in *TMEM106B* as the main susceptibility loci for frontotemporal lobar degeneration with TAR DNA binding protein (TDP-43) inclusions (FTLD-TDP) [36]. Their top SNP (rs1990622) reached a *p* value of 1.08 × 10^−11^ with an odds ratio of 0.61. They further showed that the T risk allele of rs1990622 was associated with higher mRNA levels of *TMEM106B,* but these results could not be confirmed in subsequent replication studies [37,49].

Our results show a genome-wide significant association between rs1990622 and TMEM106B protein levels (Figure 3). These results are in line with TMEM106B protein levels being modulated by the coding variant rs3173615 (T185S, Figure 4a), which is in full linkage disequilibrium (LD) with original rs1990622 [50]. Figure S1 shows a LD plot of a 14 kb region including *TMEM106B* top SNPs (circled). These results from the ROSMAP cohort confirmed the strong LD between rs1990622 and the coding variant rs3173615.

Since 2010, *TMEM106B* variants have been associated with several neurodegenerative diseases, as summarized in Table 1. More recently, the A allele of rs13237518, another SNP in strong LD with rs3173615, was identified as a new AD protective variant in a meta-analysis comprising 111 326 clinically diagnosed/“proxy” AD cases and 677 663 controls (*p* = 4.9 × 10^−11^; OR = 0.96) [34]. In the ROSMAP cohort, the A allele of rs13237518 has a frequency of 45.95% in unaffected individuals and 40.93% when combining individuals with mild cognitive impairment and Alzheimer’s disease (*p* = 0.04; OR = 0.81).

Another recent study identified a module with myelination/lysosomal genes that is upregulated in the presence of the rs1990622 T allele [51]. This module was associated with a limbic-predominant age-related TDP-43 encephalopathy neuropathological change (LATE-NC), where *TMEM106B* and *APOE*/Aβ pathways are involved [51]. These results suggest a link between TMEM106B and APOE, as shown by our initial analysis (Figure 1). Both TMEM106B and APOE might be involved in the development and progression of LATE, likely through their effects on TDP-43 pathology and other underlying mechanisms of neurodegeneration.

Initially, we hypothesized that *TMEM106B* and *APOE* might be regulated similarly at the mRNA level. The analysis of *trans*-eQTL reveals different results for *TMEM106B* mRNA levels (Figure 2). Intriguingly, the TMEM106B gene expression (Figure 2) and protein levels (Figure 3) are regulated by distinct loci. It is possible that the threonine to serine amino acid change influence the stability of the protein, modulating its degradation as suggested by Nicholson et al. [50].

Protein–protein interactions between TMEM106B and APOE seem unlikely since APOE protein levels are not influenced by T185S (Figure S2). TMEM106B is mainly expressed in neurons, while APOE is primarily found in astrocytes and microglia, indicating different cellular compartments. Interestingly, the *tmem106b* deficiency in mice was shown to dysregulate the microglial proliferation and survival in response to demyelination most likely via TREM2 reduction, whereas the *TMEM10B* risk allele rs1990622 was shown to be associated with myelin loss and a decreased microglial number in the human brain [52]. Yet, a positive correlation is observed when TMEM106B protein levels are contrasted with *APOE* mRNA levels (Figure S3).

The presence of the coding variant T185S in humans could be the reason for the similar influence on TMEM106B protein and *APOE* mRNA levels (Figure 4) in the glial compartment. These results also suggest that the interplay between TMEM106B and APOE potentially involves mechanisms related to lipid metabolism, protein clearance and neuronal health.

As the link between TMEM106B and APOE is still unclear, so is the role of TMEM106B in AD and other neurodegenerative conditions. For this purpose, we compared TMEM106B mRNA and protein levels under different conditions. In Figure 5, *TMEM106B* mRNA levels measured in temporal cortices were significantly higher in AD compared to PSP or CU.

Both Alzheimer’s disease and progressive supranuclear palsy are characterized by the presence of cytoplasmic ubiquitin-positive inclusions. Those inclusions, termed neurofibrillary tangles (NFT), differ between these two diseases in both distribution and composition, being more abundant in progressive supranuclear palsy [53]. However, AD neurons are also characterized by ubiquitin-positive inclusions in lysosome-related structures, probably explaining why *TMEM106B* levels are elevated in this condition [14].

In Alzheimer’s disease, TMEM106B protein levels increased shortly after the emergence of cognitive impairment (Figure 6). Even without dementia, amyloid plaques alone can cause a rise in *TMEM106B* mRNA levels (Figure 5, PA). In order to examine hippocampal TMEM106B in response to the remote entorhinal neurodegeneration without plaques and NFT pathologies, we employed the ECL mouse model.

Earlier studies of *Apoe* gene regulation in the ECL rodent model showed a downregulation of mRNA levels in the deafferentation phase followed by upregulation during the reinnervation phase [54]. Our study of *Tmem106b* regulation in the ECL mouse model shows a very similar expression profile (Figure S4). These results suggest that *Tmem106b* is locally regulated in response to the deafferentation and subsequent compensatory sprouting in absence of Tau and amyloid pathologies in wild-type animals.

## 5. Conclusions

Elevated levels of *TMEM106B* in mild cognitive impairment, Alzheimer’s disease and during hippocampal reinnervation in rodents all point to an active response to tissue damage that is consistent with compensatory synaptic and terminal remodeling. The associations of *TMEM106B* SNPs with the *APOE* gene expression and several neurodegenerative diseases also point in that direction.

This study is limited to cortical brain regions and should be extended to other brain regions. Future work should focus on molecular mechanisms linking TMEM106B to APOE and their specific roles in lysosome trafficking in the presence of active neurodegeneration.

## Figures and Tables

**Table 1:** Top TMEM106B pQTL SNPs and their associations with different neuropathological conditions.

SNP	Location	Disease	Ref.
rs3173615	Exon 6	FTL-TDP, CTE, PD	[30,31,32,33]
	(T185S)		
rs13237518	intron	AD, T2D	[34,35]
rs1548884	3′UTR		
rs1990622	downstream	FTLD-TDP, ALS, AD, PD, HipScl	[33,36,37,38,39,40,41,42,43,44,45,46]

Abbreviations: FTLD-TDP—frontotemporal lobar degeneration with TAR DNA binding protein inclusions; CTE—chronic traumatic encephalopathy; PD—Parkinson’s disease; AD—Alzheimer’s disease; T2D—type 2 diabetes; ALS—amyotrophic lateral sclerosis; HipScl—hippocampal sclerosis.

## Data Availability

Gene expression and proteomics data from ROSMAP and the Mayo Clinic are available for download at the AMP-AD Knowledge Portal https://adknowledgeportal.synapse.org.

## Supplementary Materials

The following supporting information can be downloaded at: https://www.mdpi.com/article/10.3390/genes15040416/s1, Table S1: Demographics; Figure S1: LD plot of a 14 kb region spanning *TMEM106B* gene locus; Figure S2: Influence of TMEM106B coding variant T185S in the dorsolateral prefrontal cortex from ROSMAP individuals; Figure S3: Correlation between TMEM106B protein levels and *APOE* mRNA levels in the dorsolateral prefrontal cortex from ROSMAP individuals; Figure S4: *Tmem106b* mRNA levels in the ECL mouse model at different post-lesion time points.
