# Supplementary material for: Association of TMEM106B with Cortical APOE Gene Expression in Neurodegenerative Conditions
Source: Genes (Basel). 2024 Mar 26;15(4):416. doi: 10.3390/genes15040416 (PMC11049136; doi:10.3390/genes15040416)
Supplement: Supplementary file 1 [file genes-15-00416-s001.zip › genes-2926629-supplementary.pdf]

Supplementary material

Supplementary Table S1. Demographics

	CU	PSP	PA	MCI	AD
**Mayo – RNAseq (n)**	**74**	**82**	**30**	**-**	**80**
Females (%)	48.6	40.2	56.7	-	61.3
APOE4 (%)	12.2	14.6	33.3	-	52.6
Age at death (median)*	86.0	74.0	86.5	-	85.0
**ROSMAP – microarray (n)**	**146**	**-**	**-**	**103**	**156**
Females (%)	63.0	-	-	61.2	59.6
APOE4 (%)	15.8	-	-	26.2	36.5
Age at death (median)*	86.2	-	-	88.7	89.6
PMI (mean)	7.9	-	-	6.9	6.1
**ROSMAP – TMT (n)**	**152**	**-**	**-**	**90**	**110**
Females (%)	70.4	-	-	66.7	77.3
APOE4 (%)	18.4	-	-	20.0	23.6
Age at death (median)*	88.0	-	-	89.9	90+
PMI (mean)	8.4	-	-	8.1	7.9

CU = Cognitively Unimpaired; PSP = Progressive Supranuclear Pasly; PA = Pathological Aging; MCI = Mild Cognitive Impairment; AD =Alzheimer’s Disease; PMI = Post-Mortem Interval (hours). *Participants with age at death over 90 years old were identified as 90+.

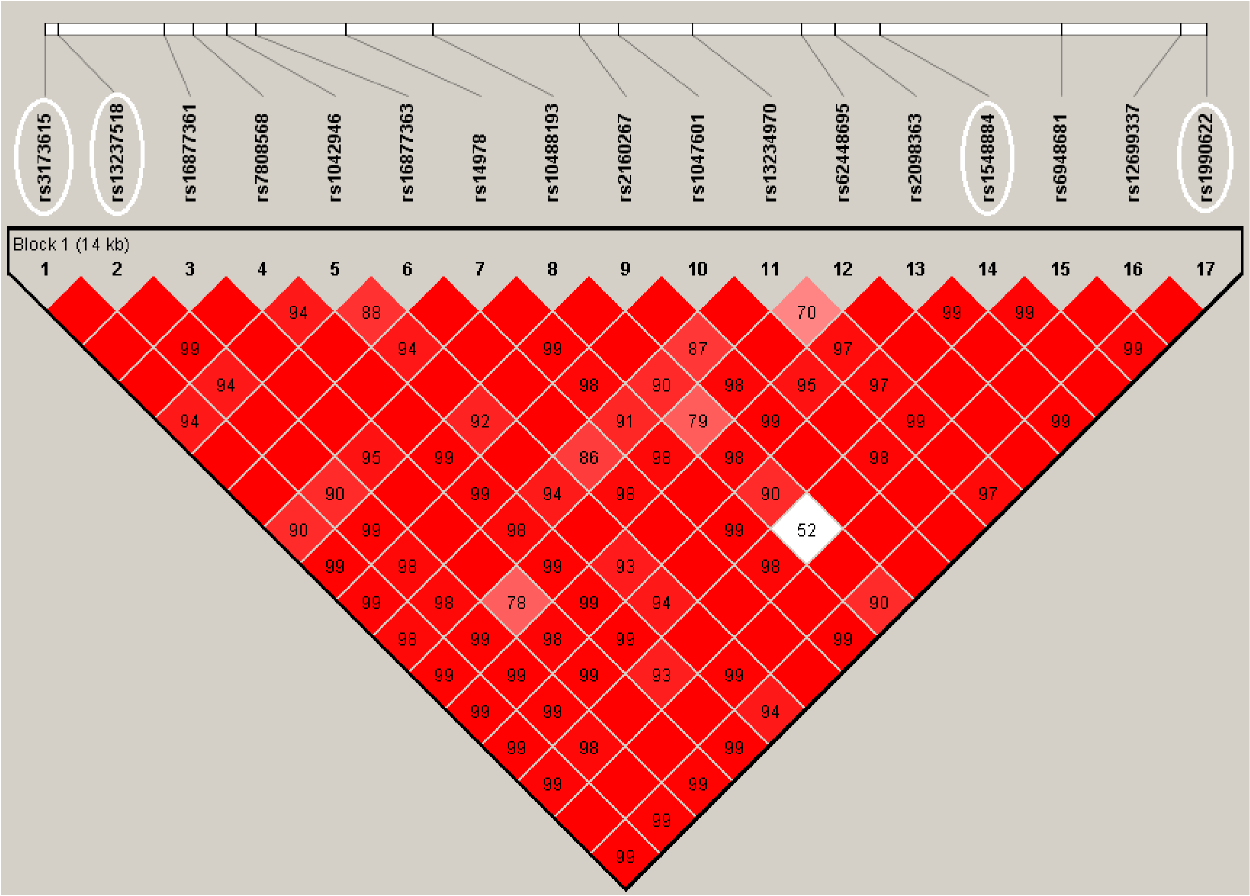

Supplementary Figure S1. LD plot of a 14 kb region spanning *TMEM106B* gene locus. Quality controlled ROSMAP genomic data were imputed as described in the material and methods section. All SNPs with a minor allele frequency below 5% were removed. Due to high LD in *TMEM106B* gene region, a variant inflation factor (r^2) threshold of 0.95 was applied using PLINK. SNPs from Table I, circled in white, were added to the pruned data. The LD plot was visualized with the help of Haploview software from Broad Institute.

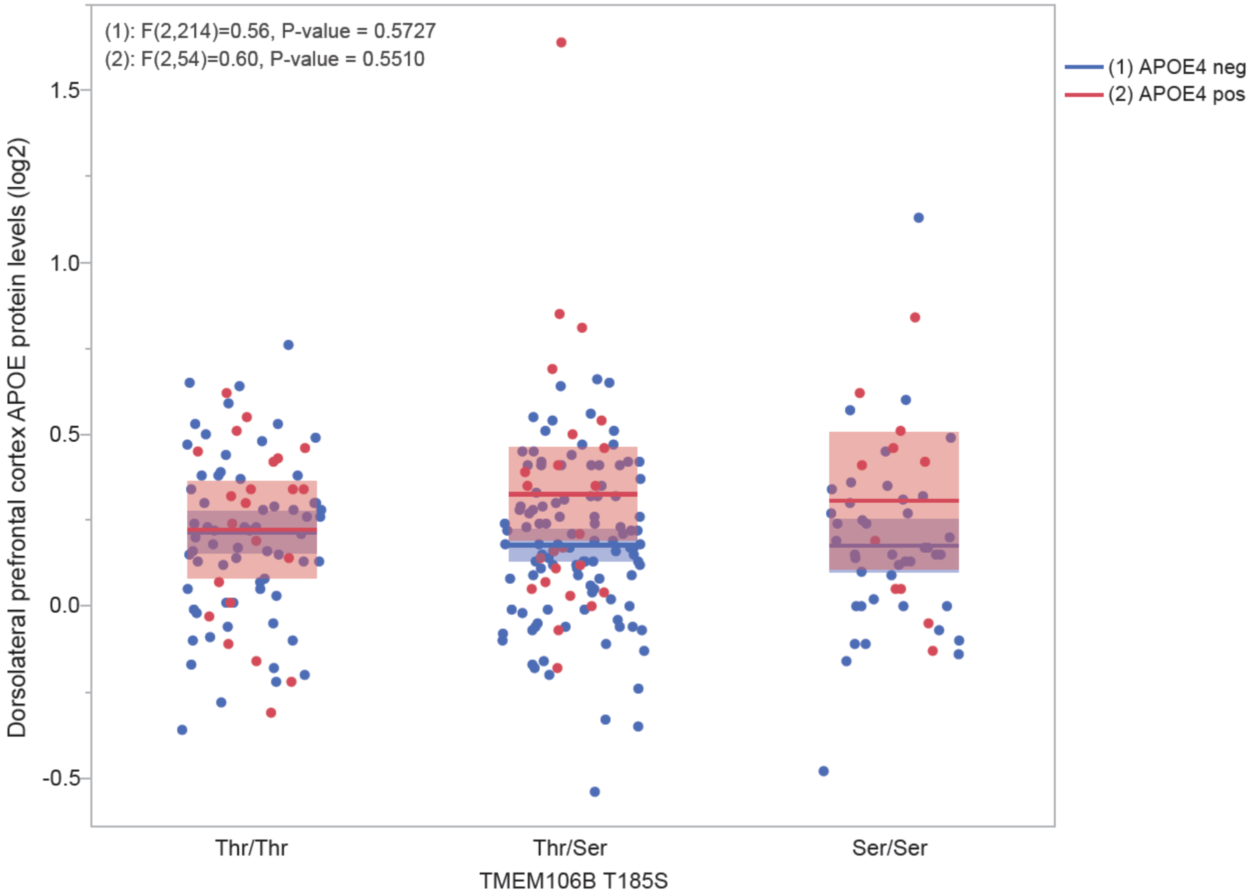

Supplementary Figure S2. Influence of TMEM106B coding variant T185S in the dorsolateral prefrontal cortex from ROSMAP individuals. APOE protein levels from 274 dorsolateral prefrontal cortices were made available by ROSMAP using tandem mass tag proteomics. APOE protein levels are not influenced by the presence of 185S amino acid in both *APOE4* negative (blue) and *APOE4* positive (red) individuals.

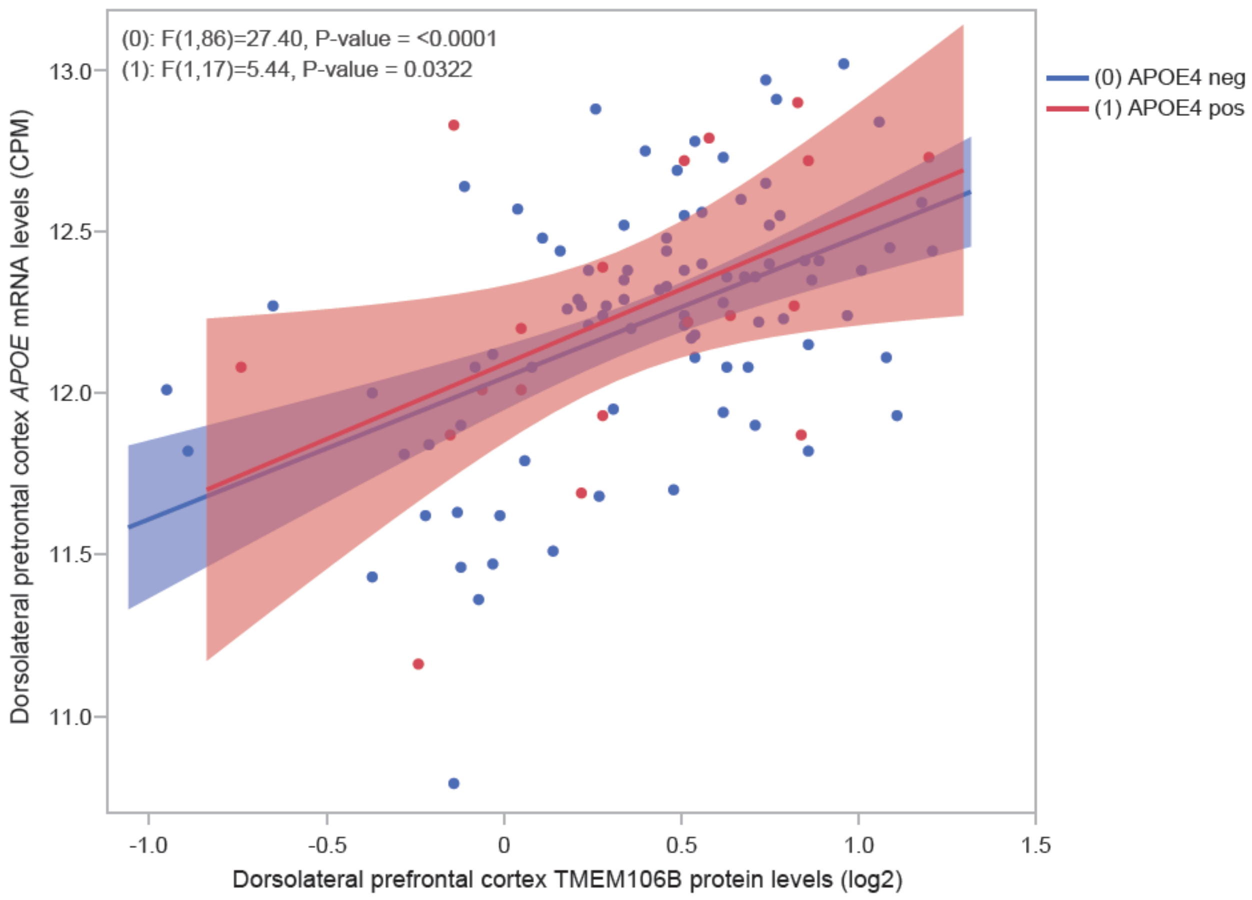

Supplementary Figure S3. Correlation between TMEM106B protein levels and *APOE* mRNA levels in the dorsolateral prefrontal cortex from ROSMAP individuals. TMEM106B protein levels correlate with *APOE* mRNA levels in both *APOE4* negative (blue) and *APOE4* positive (red) individuals.

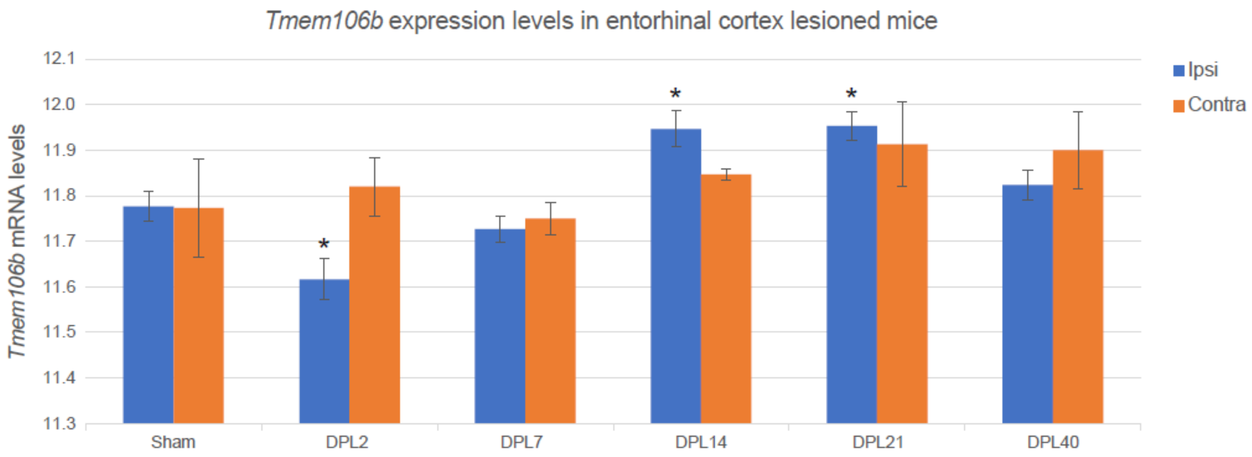

Supplementary Figure S4. *Tmem106b* mRNA levels in the ECL mouse model at different post-lesion time points. Unilateral electrolytic lesions to the entorhinal cortex were conducted on two-to-three months old male C57BL/6j mice by stereotaxic surgery. Mice were sacrificed 2, 7, 14, 21 and 40 days post-lesion (DPL) in groups of six. Total RNA was extracted from frozen ipsilateral and contralateral hippocampi using the RNeasy Lipid Tissue Mini Kit (Qiagen, Hilden, Germany). RNA quality was assessed at McGill University and Génome Québec Innovation Centre. RNA samples all had RIN > 7.8 and 260/280 ratios > 2.1. *Tmem106b* mRNA levels were measured with the Mouse Clariom^{TM} D Assay. Ipsilateral hippocampi showed reduced *Tmem106b* levels in the deafferentation phase (DPL2: p=0.046) and reached maximum levels during the reinnervation phase (DPL14: p=0.030 and DPL21: p=0.019), compared to sham-operated animals. Mean ±SEM are presented for 6 animals per time point. *p<0.05
